# A comparison of experience-dependent locomotory behaviors and biogenic amine neurons in nematode relatives of *Caenorhabditis elegans*

**DOI:** 10.1186/1471-2202-11-22

**Published:** 2010-02-19

**Authors:** Laura Rivard, Jagan Srinivasan, Allison Stone, Stacy Ochoa, Paul W Sternberg, Curtis M Loer

**Affiliations:** 1 Dept of Biology, University of San Diego, 5998 Alcala Park, San Diego, CA 92110, USA; 2 Howard Hughes Medical Institute, Division of Biology, California Institute of Technology, 1200 East California Boulevard, Pasadena CA 91125, USA

## Abstract

**Background:**

Survival of an animal depends on its ability to match its responses to environmental conditions. To generate an optimal behavioral output, the nervous system must process sensory information and generate a directed motor output in response to stimuli. The nervous system should also store information about experiences to use in the future. The diverse group of free-living nematodes provides an excellent system to study macro- and microevolution of molecular, morphological and behavioral character states associated with such nervous system function. We asked whether an adaptive behavior would vary among bacterivorous nematodes and whether differences in the neurotransmitter systems known to regulate the behavior in one species would reflect differences seen in the adaptive behavior among those species. *Caenorhabditis elegans *worms slow in the presence of food; this 'basal' slowing is triggered by dopaminergic mechanosensory neurons that detect bacteria. Starved worms slow more dramatically; this 'enhanced' slowing is regulated by serotonin.

**Results:**

We examined seven nematode species with known phylogenetic relationship to *C. elegans *for locomotory behaviors modulated by food (*E. coli*), and by the worm's recent history of feeding (being well-fed or starved). We found that locomotory behavior in some species was modulated by food and recent feeding experience in a manner similar to *C. elegans*, but not all the species tested exhibited these food-modulated behaviors. We also found that some worms had different responses to bacteria other than *E. coli*. Using histochemical and immunological staining, we found that dopaminergic neurons were very similar among all species. For instance, we saw likely homologs of four bilateral pairs of dopaminergic cephalic and deirid neurons known from *C. elegans *in all seven species examined. In contrast, there was greater variation in the patterns of serotonergic neurons. The presence of presumptive homologs of dopaminergic and serotonergic neurons in a given species did not correlate with the observed differences in locomotory behaviors.

**Conclusions:**

This study demonstrates that behaviors can differ significantly between species that appear morphologically very similar, and therefore it is important to consider factors, such as ecology of a species in the wild, when formulating hypotheses about the adaptive significance of a behavior. Our results suggest that evolutionary changes in locomotory behaviors are less likely to be caused by changes in neurotransmitter expression of neurons. Such changes could be caused either by subtle changes in neural circuitry or in the function of the signal transduction pathways mediating these behaviors.

## Background

Animals use their nervous systems to sense and respond dynamically to changing environments. Nematodes constitute one of the most diverse and populous phyla in the animal kingdom, with estimates of up to 1 million extant species [1]. Although all nematodes share a similar basic body plan, they have distinct morphological adaptations and can differ in length by four orders of magnitude. They have a wide geographical distribution, exploit diverse ecological niches, and can survive extreme environments like the Antarctic [2]. Nematodes are both parasitic and free-living and can obtain nutrients from a wide variety of materials. Additionally, nematodes have evolved several different reproductive strategies, exhibiting gonochorism (male-female), hermaphroditism, heterogony, and parthenogenesis [3]. The phylum Nematoda also exhibits genomic diversity. An analysis of expressed-sequence tags from 30 different species revealed that 30-50% of the sequences studied were unique to individual species [4,5].

The detailed molecular genetic and neuronal bases of many nematode behaviors such as egg laying [6,7], mechanosensation [8], pharyngeal-pumping [9], and male-mating [10] have been described in detail from *C. elegans*. Intraspecific variation in behaviors has also been examined in *C. elegans *and other nematode species, as well as differences between related species. For example, some wild isolates of *C. elegans *aggregate and feed socially whereas other strains disperse and forage independently [11]. Species from two clades of entomopathogenic nematodes, Steinernematidae and Heterorhabditidae, exhibit different behaviors associated with infection of host insects [12]. Four closely related species of *Pristionchus *have unique chemoattraction profiles to 11 compounds classified as insect pheromones or plant volatiles [13]. Finally, males of two gonochoristic *Caenorhabditis *species, *C. remanei *and *C. brenneri*, are more efficient at spicule insertion during mating than males of the hermaphroditic species *C. briggsae *and *C. elegans *[14].

Few studies, however, have attempted to address the neural control of behavior across different species of nematodes [15,16]. Such a study first requires the identification of a behavior that is at least partially conserved in multiple species. Then, the neural circuitry of the selected species can be examined.

We undertook an interspecific comparison of a locomotory behavior modulated by feeding. Sawin and colleagues [17] showed that the presence or absence of food (bacteria) as well as feeding status (well-fed or starved) affects the rate of locomotion of *C. elegans*. Well-fed *C. elegans *worms have the fastest rate of locomotion in the absence of food, and their locomotion slows when food is present. This behavior is known as the "basal slowing response" (BSR). Worms recently deprived of food move even more slowly in food, exhibiting a behavior known as the "enhanced slowing response" (ESR). The neural circuits that control the basal and enhanced slowing responses are distinct [17]. The BSR is mediated by the dopaminergic CEP, ADE, and PDE neurons, which have sensory endings in the cuticle and likely detect the presence of bacteria by a mechanical stimulus. The ESR is mediated by serotonin. Mutant strains lacking serotonin have a defective ESR that can be rescued by the addition of exogenous serotonin [17]. Fluoxetine, a selective serotonin reuptake inhibitor, potentiates the ESR, while serotonin antagonists prevent the behavior [17]. Ablation experiments have failed, however, to unambiguously identify the serotonergic neurons required for the ESR [17].

We scored several species of nematodes whose phylogenetic relationships to *C. elegans *are known for the presence of the basal and enhanced slowing responses. We examined the patterns of dopamine-containing neurons in these species, and then also investigated whether the ESR was modulated by serotonin and what serotonergic neurons are present. We found that only some of the species examined had slowing responses under the conditions tested, and there was no stereotypical array of serotonergic neurons present that might be required for the ESR. Dopamine-containing neurons were highly conserved although the BSR under the conditions tested was not. We propose the evolutionary source of the slowing behaviors based on a nematode phylogeny.

## Results

### Phylogenetic relationships of the nematode species tested for food-modulated behaviors

**Figure 1:**
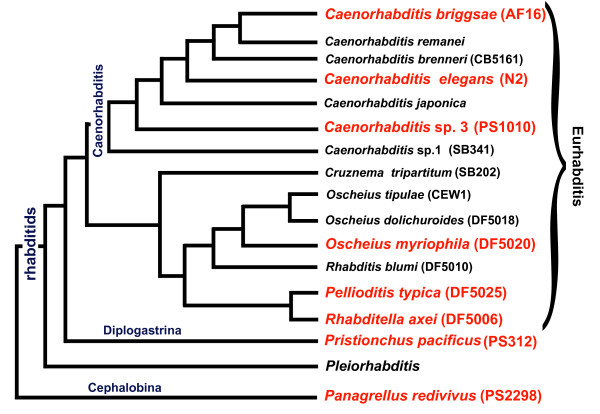
**Phylogenetic tree of rhabditid nematodes used in our comparative study of modulated behaviors**. The tree is congruent with trees from Kiontke and Fitch [3], based on SSU and LSU rRNA genes and RNAPII large subunit gene sequences. Species shown in red were analysed in this study for food-modulated behaviors and patterns of dopaminergic and serotonergic neurons.

We chose seven representative free-living species from different groups of rhabditids with a range of phylogenetic distances to *C. elegans *for our comparative analysis. All species selected exhibited a sinusoidal pattern of body bends similar to *C. elegans*. The species tested were *C. elegans *(N2), *C. briggsae *(AF16), *Caenorhabditis *sp. 3 (PS1010), *Oscheius myriophila *(DF5020), *Pellioditis typica *(DF5025), *Rhabditella axei *(DF5006), *Pristionchus pacificus *(PS312), and *Panagrellus redivivus *(PS2298) (Figure 1). Based on current molecular data, rhabditids can be divided into two major clades: Eurhabditis and Pleiorhabditis [3]. The species include both gonochoristic and hermaphroditic life histories, so hermaphrodites or females were used. *C. elegans, *along with other members of the *Caenorhabditis *genus, belongs to the Eurhabditis clade [3]. From the genus *Caenorhabditis*, we chose *C. elegans *(N2), *Caenorhabditis briggsae *(AF16) and the less closely related *Caenorhabditis *sp. 3 (PS1010) [3,18]. *C. elegans *and *C. briggsae *were isolated from compost, and *Caenorhabditis *sp. 3 (PS1010) was isolated from galleries of palm and sugarcane weevils [19]. *Oscheius myriophila *(DF5020), *Pellioditis typica *(DF5025) and *Rhabditella axei *(DF5006), also belong to the Eurhabditis clade, but belong to a different branch than *Caenorhabditis *[3]. From the group of diplogastrids, we chose the satellite model system *Pristionchus pacificus *(PS312) [20,21], which belongs to a genus that associates with beetles [22]. *Panagrellus redivivus *(PS2298) was chosen as an outgroup. This nematode species has been isolated from sugar-rich environments such as sap of rubber trees and brewery mash [23] (Figure 1).

### Some nematode species do not exhibit basal and enhanced slowing responses

**Figure 2:**
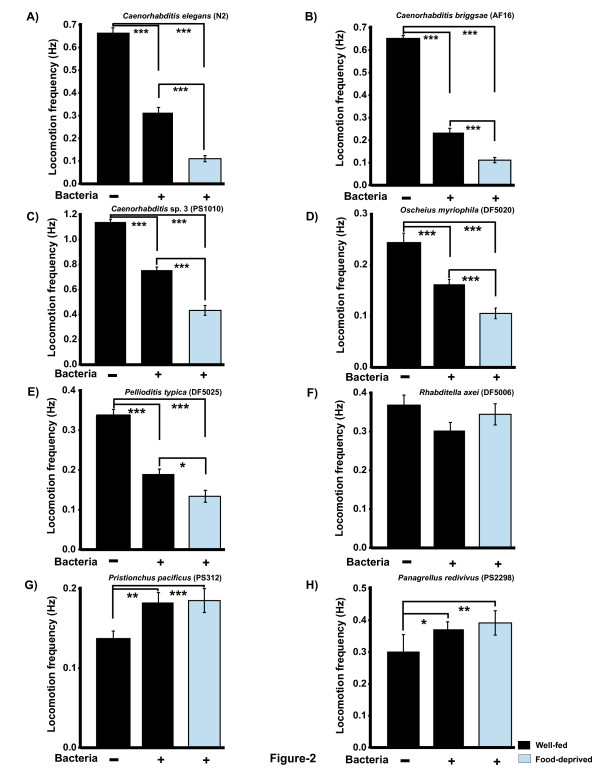
**Locomotory rates of well-fed and food deprived animals both on and off bacteria**. In all species, the baseline locomotory rate or frequency (mean ± SEM) is represented by the first column and was calculated for well-fed worms transferred to an empty agar plate (no bacteria). Second column - locomotory rate for well-fed worms transferred to a bacterial lawn. Third column - locomotory rate for previously food-deprived worms transferred to a bacterial lawn. (A-E) *C. elegans, C. briggsae, Caenorhabditis *sp. 3, *O. myriophila, *and *Pellioditis typica *all exhibited basal and enhanced slowing responses. For all these species, there were statistically significant differences among the groups by 1-factor ANOVA (P << 0.001). Asterisks indicate statistically significant differences in planned pairwise comparisons between 'no food' and '+bacteria (well-fed)' [asterisks over this column] or 'no food' and '+bacteria (food-deprived)' *P < 0.05, ** P < 0.01, *** P < 0.001. (F-H) *R. axei, Pristionchus pacificus *and *Panagrellus redivivus *did not exhibit either basal slowing or enhanced slowing responses. There were no statistically significant differences among the groups (1-factor ANOVA, P > 0.05) for *R. axei. Panagrellus *and *Pristionchus *moved significantly faster on bacteria than off bacteria, but there was no significant difference between well-fed or food-deprived worms (P > 0.05). (A-F) Manual counting of body bends was used [17]. The numbers of worms tested varied in each column: *C. elegans *(n = 60-72); *C. briggsae *(n = 87-109), *C*. sp. 3 (n = 54-80), *O. myriophila *(n = 139-146), *P. typica *(n = 101-145), *R. axei *(n = 62-89) (G, H) Automated tracker scoring of locomotory rate was used. Number of worms scored per column: *P. pacificus *(n = 14-17), *P. redivivus *(n = 21). Both manual and automated tracker methods were used to quantify locomotory rate for all species other than *Pristionchus pacificus *and *Panagrellus redivivus *(automated only) and *Pellioditis typica *(manual only); results were comparable and yielded the same statistical significance.

We used both manual and automated methods to determine the locomotory behavior of eight species of nematodes chosen in our analysis (see Materials and Methods for details, and Additional file 1, Figure S1). If the basal and enhanced slowing responses (BSR and ESR) present in *C. elegans *are adaptive, it is reasonable to predict that other species will also exhibit the behaviors. To explore the conservation of the slowing behaviors, we first determine a baseline locomotory rate for each species using well-fed animals on an agar plate with no bacteria (Figure 2A-H). Rates of locomotion varied considerably between species. *Caenorhabditis*sp. 3 had the highest average locomotory rate at 1.14 ± 0.02 Hz (body bends per second, mean ± SEM). *Pristionchus pacificus *had the lowest locomotory rate at 0.13 ± 0.01 Hz. As previously reported [17], well-fed *C. elegans *slowed significantly (basal slowing/BSR) in the presence of bacteria, and starved animals slowed even more dramatically (enhanced slowing/ESR) following reintroduction to bacteria (Figure 2A). Four other species, *C. briggsae, Caenorhabditis *sp. 3, *O. myriophila *and *Pellioditis typica*, exhibited both a BSR and ESR similar to that seen in *C. elegans *(Figure 2B-E). For well-fed *R. axei *no BSR was detectable (Figure 2F). Similarly, the locomotory rate of starved *R. axei *on bacteria did not differ significantly from the baseline locomotory rate (Figure 2F). Both *Pristionchus pacificus *and *Panagrellus redivivus *lacked a BSR or ESR; the locomotory rate was significantly higher in the presence of bacteria in both well-fed and starved animals (Figure 2G,H). This observation is exactly opposite to the locomotory behavior of *C. elegans, *wherein the worm's locomotory rate slows in the presence of food.

**Figure 3:**
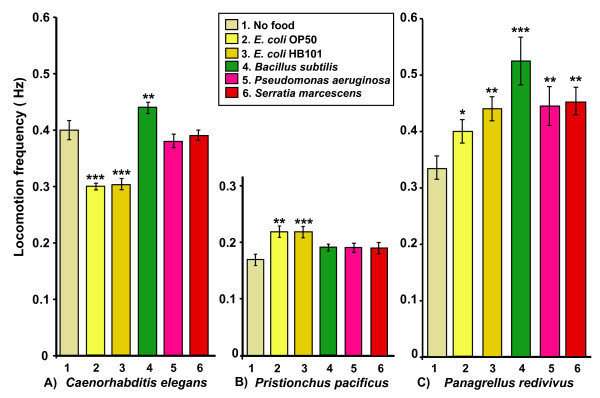
**Locomotory rates of worms on different bacterial strains**. Well-fed *C. elegans *(A), *Pristionchus pacificus *(B) and *Panagrellus redivivus *(C) were tested for locomotion on five different bacterial strains vs. no bacteria. Histograms show mean ± SEM locomotion frequency measured using an automated tracker. Locomotion frequencies for each nematode species were compared using 1-factor ANOVA followed by planned pairwise comparisons. For each species, there were statistically significant differences among the groups (P << 0.001). We performed planned pairwise comparisons of 'no bacteria' vs. each other bacterial strain. Statistically significant differences in pairwise comparisons with 'no food' are indicated over the each column compared [* - P < 0.05, ** - P < 0.01, *** - P < 0.001]. Number of worms scored per column: *C. elegans *(n = 28-50), *P. pacificus *(n = 13-16), *P. redivivus *(n = 11-21).

It is possible that in species lacking a BSR or ESR in our assays, the food presented (*E. coli*) was not adequate to elicit a response. Therefore, we tested two of the species - *Pristionchus pacificus *and *Panagrellus redivivus *- for their basal locomotion on other potential food sources (Figure 3). We chose some bacteria that were gram-positive and some gram-negative (see Materials and Methods). We also tested *C. elegans *with the same set of bacteria. *C. elegans *slowed significantly on either *E. coli *strain tested, but not on the other three strains (*Bacillus subtilis, Pseudomonas aeruginosa, *and *Serratia marcescens*); in fact, worms moved significantly faster on *B. subtilis *(Figure 3A). *Pristionchus pacificus *showed a significant increase in locomotory rate on the *E. coli *strains OP50 and HB101, but did not change significantly on the other bacteria (Figure 3B). *Panagrellus redivivus *moved significantly faster on all the bacterial strains tested (Figure 3C).

### Dopaminergic neurons are similar across nematode species

**Figure 4:**
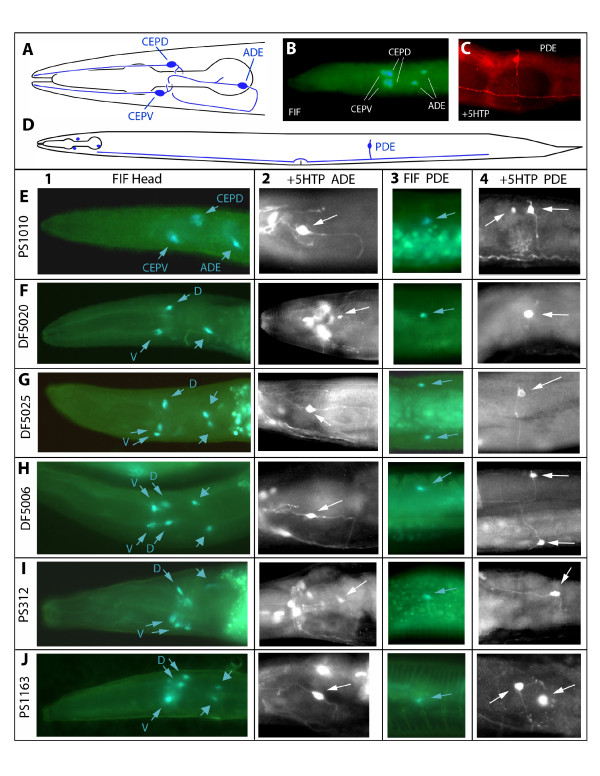
**Dopaminergic neurons in free-living Rhabditid nematodes**. A. Schematic of *C. elegans *head showing dopaminergic (DA) neurons on one side; each neuron has a bilaterally symmetric partner. CEPD: dorsal cephalic neuron; CEPV: ventral cephalic neuron; ADE: anterior deirid neuron. In all images, anterior is to the left, and dorsal up (except as noted). B. FIF-treated *C. elegans *larval head showing DA neurons (dorsoventral view) on right and left sides. C. PDE (posterior deirid neuron) neuron in lateral, mid-posterior body wall of *C. elegans *treated with 5HTP and stained with anti-serotonin (TRITC-conjugated secondary antibody). A dorsally directed dendrite arises from the soma: an axon extends ventrally and projects within the ventral nerve cord. D. Schematic of *C. elegans *body showing locations of DA neurons, including the PDE. Only the somata are shown for the head DA neurons. E-J. Dopaminergic neurons in species we examined, revealed by FIF and immunofluoresence. The pattern of DA cells in *C. briggsae *is identical to that of *C. elegans *(data not shown). Column 1 (FIF Head): FIF-treated worm heads. Slender-headed arrows indicate presumptive CEPD and CEPV homologs (in F-J, abbreviated D or V). Broad arrowheads indicate presumptive ADE homologs. In some panels both right and left cells can be seen (more than one arrow). Column 2 (+5HTP ADE): Putative ADE homologs visualized with anti-serotonin in 5HTP-treated worms (somata indicated with arrow). Soma locations and processes are quite similar to those in *C. elegans*, although many ADE posterior-directed neurites leading to commissures are longer than illustrated for *C. elegans*. Some panels are montages of nearby planes of focus to better show the cell morphology. Column 3 (FIF PDE): Lateral, mid-posterior body wall of FIF-treated worms showing somata of likely PDE homologs (arrow); in some panels both right and left cells can be seen (more than one arrow). Strong gut fluorescence often makes PDEs difficult or impossible to see by FIF. Column 4 (+5HTP PDE): PDE homologs in body wall visualized with anti-serotonin in 5HTP-treated worms (soma indicated by arrow); in some panels both right and left cells can be seen (more than one arrow), although sometimes the soma is out of the plane of focus. E. *Caenorhabditis *sp. 3 (PS1010). F. *Oschieus myriophila *(DF52020). G. *Pellioditis typica *(DF5025). Panel G3 (FIF PDE) is a ventral view showing both left and right PDE somata. H. *Rhabditella axei *(DF5006). Panel H4 (+5HTP PDE) is a ventral view in which both left and right cells can be seen. I. *Pristionchus pacificus *(PS312). In panel I4 (+5HTP PDE), note the long anteriorly directed PDE dendrite. J. *Panagrellus redivivus *(PS1163).

Dopamine has been implicated in the control of the BSR in *C. elegans*. Unlike wild-type animals, worms with highly reduced dopamine levels such as *cat-2 *mutants [24,25], fail to slow in bacteria [17]. Elimination of a specific dopamine receptor, *dop-3*, expressed in ventral cord motoneurons, also eliminates the BSR [26]. To determine whether the correlation between dopamine production and the BSR is conserved across species, we examined the pattern of dopamine-containing neurons in the same nematode species used in our behavioral studies. We used formaldehyde-induced fluorescence (FIF) and 5HTP-induced serotonin immunoreactivity in DA neurons to corroborate FIF staining and to observe neuronal morphology (see Figure 4 legend and below for explanation). *C. elegans *hermaphrodites have 4 bilateral pairs of dopaminergic neurons located in cephalic, deirid and postdeirid sensilla: 4 CEPs, 2 ADEs, and 2 PDEs (Figure 4A-D, [25]). All of these neurons are probably mechanosensory, as they extend ciliated dendrites with endings embedded in the cuticle, and do not contact the external environment [27]. CEP endings are at the tip of the 'nose,' and ablation of all 4 CEP neurons results in a moderately reduced BSR [17]. ADE and PDE have endings in the cuticle in the anterior and posterior body, respectively, and ablation of either cell type alone does not affect the BSR [17]. Combined ablation of all 8 dopaminergic neurons completely eliminates the BSR, indicating that the CEPs, ADEs, and PDEs all contribute to the behavior [17]. The BSR can be induced with 20-50 μm particles (Sephadex G-200 beads), supporting the mechanosensory vs. chemosensory nature of the cells' function [17].

We observed that all seven species had FIF-positive somata that are plausible homologs of the bilaterally symmetric head cephalic sensory neuron CEPs, the anterior deirid neuron ADEs and postdeirid PDEs (Figure 4E-J). In all the species examined, presumptive ventral CEPs (CEPV) were slightly more anterior than dorsal CEPs (CEPD), just as in *C. elegans*. The putative CEP neurons were more strongly and reliably stained by FIF; in some species, we saw putative ADE neurons less often and PDE neurons infrequently. This may partly be due to the very high background fluorescence in the body, especially from the intestine. There is typically much less background in the head and tail. Putative ADEs were located in the posterior head around the posterior bulb of the pharynx, although in some species these cells were further anterior than typically found in *C. elegans*. Along the dorsoventral axis, ADE somata were located laterally to sublaterally. Putative PDE homologs were located subdorsally, and mid-posterior along the anteroposterior axis of the body.

For the species examined, we saw that FIF staining worked better in larvae than adults. We consistently observed 3 pairs of cell bodies in the head of the larvae, indicating little or no change in DA cells postembryonically other than the appearance of the PDE. The PDE neurons are born postembryonically in mid-late L2 stage in both *C. elegans *and *Panagrellus redivivus *[28,29], and hence are seen only in older larvae and adults. In a few species, we occasionally found an additional FIF-positive cell in adults in the head and/or body, but these were less reliable. In *Pristionchus*, we saw what appeared to be a ventral unpaired neuron in few adult heads; in *O. myriophila *we saw a few worms with a pair of FIF-positive cells in the tail.

In our studies we found that FIF stained neuronal cell bodies, but only rarely processes. To obtain a better picture of the neuronal processes, we used serotonin antibody staining after treating worms with 5-hydroxytryptophan (5HTP), the immediate precursor to serotonin. 5HTP is taken up by both serotonergic and dopaminergic neurons and converted into serotonin by their shared AADC enzyme [30,31]. Therefore, dopaminergic neurons are stained among a background of known serotonergic neurons. With this technique, neuronal processes are often well-stained, revealing the morphology of the neurons. In stained worms where we had previously seen FIF-positive somata, we observed serotonin immunoreactive cells that were not seen without 5HTP treatment. [5HTP-stained cells must be matched with FIF-positive cells since 5HTP can also strengthen staining in weakly or variably staining serotonergic cells.] Deirid neurons (ADEs and PDEs), which are relatively isolated from other neurons (including other known serotonergic neurons), were well-stained by this technique, showing bipolar cell bodies with sensory dendrites extending toward the outer surface of the worm. The morphology of putative ADE and PDE neurons in every species was very similar to that known from *C. elegans*, with minor differences in some species (Figure 4, Columns 2 and 4 '+5HTP'). For example, the putative PDE homolog in *Pristionchus pacificus *had a longer dendrite that extended anteriorly rather than dorsally.

The heads in 5HTP-treated worms were more difficult to assess, especially in species that have numerous serotonergic neurons in the same region of the head as putative CEP neurons (see descriptions of serotonergic head neurons below). Nevertheless, we were frequently able to see 4 additional somata in the same location of FIF positive cells in the head. We also often saw 4 additional processes extending anteriorly into the 'nose' of the worms, consistent with the morphology known for *C. elegans *CEP neurons. Overall, the patterns of dopaminergic neurons in all the species we examined were nearly identical, suggesting strong conservation of this portion of the nervous system.

### Serotonergic neurons in the head differ dramatically across nematode species

**Figure 5:**
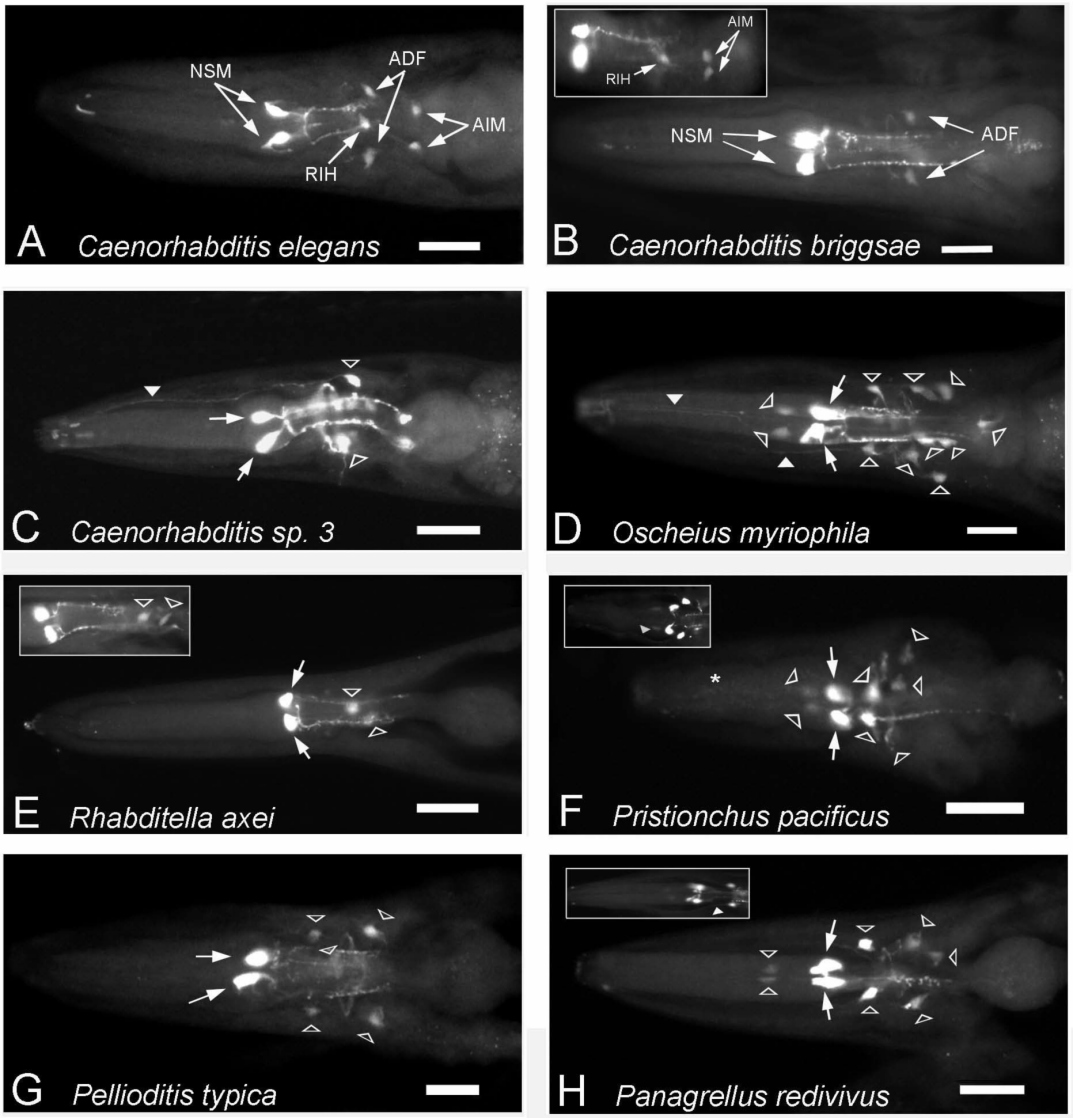
**Serotonergic head neurons in free-living Rhabditid nematodes**. All panels show serotonin immunoreactivity in whole-mount heads of adult nematodes. Anterior is to the left, ventral view. Calibration bars in each panel are 20 μm. In B-H, arrows indicate NSMs, open arrowheads indicate other serotonin immunoreactive somata, and closed arrowheads indicate serotonin immunoreactive processes. (A) *C. elegans *(N2) with labeled neurons. (B) *C. briggsae *(AF16). (C) *Caenorhabditis *sp. 3 (PS1010). The two large bright spots in the posterior bulb of the pharynx are large NSM-associated varicosities, and not somas. [Double-labeling with DAPI staining shows no nuclei are associated with these blobs.] The filled arrowhead indicates a neurite extending to the amphid associated with a putative ADF homolog. (D) *Oscheius myriophila *(DF5020). (E) *Rhabditella axei *(DF 5006). Inset: two serotonin-immunoreactive neurons posterior to the NSMs in a different *R. axei *animal. (F) *Pristionchus pacificus *(PS312). The asterisk represents the position of serotonin immunoreactive processes occasionally visible extending from a pair of neurons immediately anterior to the NSMs. Inset: a serotonin immunoreactive process extending from a paired neuron located posterior and lateral to the NSM in a different *Pristionchus pacificus *animal. (G) *Pellioditis typica *(DF5025). (H) *Panagrellus redivivus *(PS1163). Inset: a serotonin immunoreactive process extending from a paired neuron located posterior and lateral to the NSM in a different *Panagrellus redivivus *worm.

Serotonin has been implicated in the control of the ESR in *C. elegans*. Animals with mutations in genes required for serotonin biosynthesis, such as *bas-1, cat-4 *and *tph-1*, move faster than wild-type animals when starved and placed on bacteria [17,32]. Serotonin probably controls the ESR via a chloride-selective ion channel serotonin receptor (MOD-1); mutants in the *mod-1 *gene are defective in the ESR[33]. Serotonin-immunoreactive (serotonin-IR) head neurons found in the *C. elegans *hermaphrodite have been previously characterized. The first neurons identified were the NSMs or "neurosecretory motor neurons" [34], a bilaterally symmetric pair of neurons with somata located within the anterior bulb of the pharynx and bifurcating processes that extend into the pharyngeal isthmus [35]; recently the structure of the NSM has been revised to include an additional fine neurite extending posteriorly into the terminal bulb of the pharynx [36]. Ventral and lateral to the isthmus of the pharynx are the ADFs, a bilaterally symmetric pair of serotonin-IR sensory neurons (Figure 5A). ADF neurons extend processes to the nerve ring and amphid [37]. RIH is an unpaired serotonin-IR interneuron found ventral to the isthmus of the pharynx (Figure 5A) that extends processes to the nerve ring and other pharyngeal ganglia [37]. Finally, the AIMs are a bilaterally symmetric pair of interneurons with somata located near the midline just ventral to the posterior bulb of the pharynx (Figure 5A). AIM interneurons extend a single process to the nerve ring (White et al. 1986). The ADF, RIH and AIM processes are rarely detected by immunohistochemistry in *C. elegans*. It is unclear, however, which serotonin-IR head neurons mediate the ESR. Ablation of the NSMs, the most reliably and intensely staining serotonin-IR head neurons, only modestly affects the ESR, and the additional ablation of other serotonin-IR head neurons with the NSMs does not further affect the locomotion rate [17]. In serotonin-deficient *tph-1 *mutants, expression of wild type *tph-1 *in NSMs partially restores the ESR; expression in ADF neurons does not [32].

To explore which neurons might be associated with the ESR, we examined serotonin immunoreactivity in the heads of the seven nematode species used in our behavioral studies. Neurons required for the ESR may be conserved in species that also exhibit the behavior. We observed clearly identifiable NSMs with cell bodies located ventrally in the anterior bulb of the pharynx in all species studied (Figure 5A-H arrows). The NSMs had bifurcating neurites projecting through the isthmus just to the posterior bulb of the pharynx, as observed in *C. elegans*. All the species tested in our analysis showed strong serotonin immunoreactivity in putative NSM neurons. [The presence of putative NSM homologs in these species has been described previously (Loer & Rivard, 2007).] We also examined other serotonin-IR head neurons compared to those found in *C. elegans*. We observed ADF-like neurons in all species except *R. axei *(Figure 5B-H, F and 5H insets). These neurons were categorized as ADF-like based on the position of their somata and occasionally a visible projection that is likely part of the amphid (Figure 5C, D, F and 5H insets closed arrowheads). Again, there was no correlation between the presence of an ADF-like neuron and the ESR. Some species contained possible AIM and RIH homologs, however, such identification would be highly tentative based on soma position alone in the absence of stained projections. Other serotonin-IR neurons that can be identified as likely homologs across species include a faintly staining bilaterally symmetric pair located within the pharynx just anterior to the NSMs, seen in *Oscheius myriophila, Pristionchus pacificus*, and *Panagrellus redivivus *(Figure 5D, F, H). These cells are not seen in *C. elegans*, and, as before, there was no correlation between the presence of these neurons and the ESR.

The number of serotonin-IR head neurons varied dramatically in the species examined. *O. myriophila *had up to thirteen serotonin-IR head neurons (Figure 5D), which was the most observed; *Caenorhabditis *sp. 3 and *R. axei *had the fewest, with four each (Figure 5C, E). It is possible, however, that the cells in *Caenorhabditis *sp. 3 match those of *C. elegans *and *C. briggsae*. We noted that the axons of the NSMs in *Caenorhabditis *sp. 3 have numerous brightly staining varicosities, which could be obscuring a faint second pair of serotonin-IR neurons. *Caenorhabditis *sp. 3 also rarely appear to have a faint unpaired cell. Among all the species, the number of serotonin-IR neurons in the head does not appear to correlate with the presence of the ESR. *Caenorhabditis *sp. 3 and *R. axei *both have only four serotonin-IR head neurons (Figure 5C, D) but *Caenorhabditis *sp. 3 exhibited an ESR, and *R. axei *did not. The staining patterns of *Pristionchus pacificus *and *Panagrellus redivivus *appear very similar to that of *C. elegans*, with the addition of the faintly staining pair of neurons anterior to the NSMs (Figure 5A, F and 5H); *C. elegans *exhibits an ESR whereas *Pristionchus pacificus *and *Panagrellus redivivus *does not. Finally, there was no correlation between the number of serotonin-IR head neurons and the overall rate of locomotion under well-fed or starved conditions. Overall, we conclude that the pattern of serotonin-IR neurons in the species studied cannot be used as an indicator of locomotory behavior.

### The enhanced slowing response is blocked by a serotonin antagonist in some, but not all species

**Figure 6:**
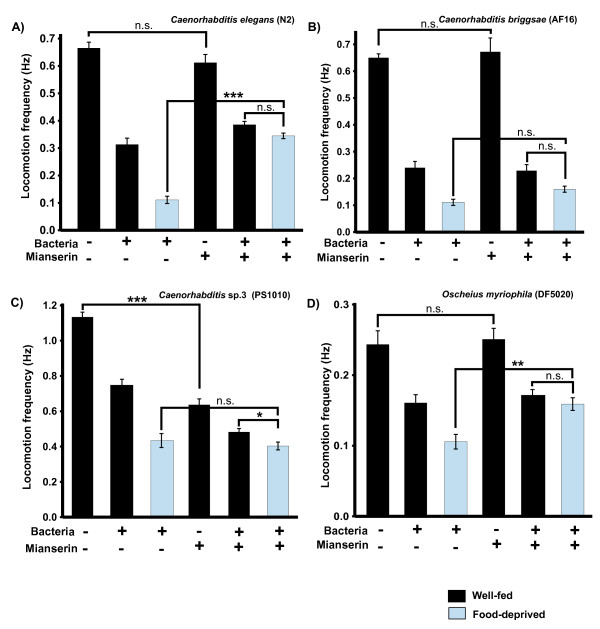
**Effect of serotonin antagonist mianserin on modulation of locomotion**. Histograms show mean ± SEM locomotion frequency from manual observations. Worms from species showing enhanced slowing responses were incubated for 30 minutes in 20 μM mianserin hydrochloride prior to testing (see Materials & Methods). Worms not treated with mianserin are the same as shown in Figure 2. (A) *C. elegans *(B) *C. briggsae *(C) *C*. sp. 3 (D) *O. myriophila*. For all these species, there were statistically significant differences among the groups by 1-factor ANOVA (P << 0.001). Selected pairwise comparisons are indicated by lines connecting columns with significance as indicated [n.s. - not significant (P > 0.05), * - P < 0.05, ** - P < 0.01, *** - P < 0.001] Mianserin significantly attenuated the enhanced slowing response in A & D, but did not affect the locomotory rate with or without bacteria. Mianserin did not affect enhanced slowing in *Caenorhabditis *sp. 3 but reduced the locomotory rate on or off bacteria. Not indicated in the figure (but presented in text) are pairwise comparisons of untreated vs. treated with mianserin, well-fed worms on bacteria (*C. elegans*, n.s.; *C. briggsae*, n.s.; *C*. sp. 3, P < 0.001; *O. myriophila*, n.s.) Numbers of worms tested in mianserin treatments: *C. elegans *(n = 58-77), *C. briggsae *(n = 34-49), *C*. sp. 3 (n = 61-68), *O. myriophila *(n = 60-85).

We used a serotonin antagonist (mianserin), to test whether enhanced slowing is mediated by serotonin in the species in which we observed the response. Although in mammals mianserin is classically an antagonist of 5-HT_2 _serotonin receptors [38], in *C. elegans *it blocks both 5-HT_2_-like receptors [39] and the MOD-1 5-HT receptor shown to mediate the ESR [33]. Animals were preincubated for 30 minutes on seeded or unseeded agar plates supplemented with 20 μM mianserin prior to locomotion assays. As previously reported [17], we also found that mianserin treatment decreased or eliminated the ESR in *C. elegans *(Figure 6A, well-fed worms on bacteria vs. food-deprived worms on bacteria, both mianserin-treated, P > 0.05; see Figure legend for details of statistics). Similarly, in our experiments, mianserin did not significantly affect locomotion either on or off bacteria (Figure 6A). We saw the same results with mianserin in *O. myriophila *- mianserin eliminated the ESR (Figure 6D), well-fed worms on bacteria vs. food-deprived worms on bacteria, both mianserin-treated, P > 0.05). Results with *C. briggsae *were less clear: although treatment with mianserin eliminated a significant difference between well-fed worms on bacteria vs. food-deprived worms on bacteria (Figure 6B, two rightmost columns, P > 0.05), there was also no significant difference between food-deprived worms on bacteria untreated vs. treated with mianserin (Figure 6B, blue columns). For both *O. myriophila *and *C. briggsae*, mianserin affected neither baseline locomotion in the absence of bacteria (P > 0.05), nor the BSR in the presence of bacteria (P > 0.05, Figure 6B and 6D).

In contrast, mianserin had a very different effect in *Caenorhabditis *sp. 3 (Figure 6C). Mianserin significantly reduced the locomotory rate both off and on bacteria (with a BSR still apparent in the presence of mianserin, P < 0.001, comparison not shown on graph), but had no effect on the locomotion of food-deprived worms on bacteria (Figure 6C, food-deprived worms on bacteria, untreated or treated with mianserin, P > 0.05). In the presence of mianserin, an ESR is also still apparent (Figure 6C, well-fed worms on bacteria vs. food-deprived worms on bacteria, both mianserin-treated, P < 0.05). One possibility is that mianserin in *C*. sp. 3 is acting more like a 5HT agonist than an antagonist, reducing the rate of locomotion overall; this could make it more difficult to detect an effect on the ESR. Clearly the effect of mianserin is altered in *Caenorhabditis *sp. 3 relative to the other species tested, and the expression patterns or specificity of 5HT receptors in *Caenorhabditis *sp. 3 seems likely to be quite different. Whatever the change, the same results were obtained in locomotory assays of *Caenorhabditis *sp. 3 preincubated in 44 μM methiothepin mesylate (data not shown); methiothepin is a serotonin antagonist that in *C. elegans *has broader specificity and also inhibits MOD-1 [33]. These preliminary pharmacology experiments are suggestive of a similar function of serotonin in mediating the ESR in *C. briggsae *and *O. myriophila*, although they must be viewed with caution given our currently limited information about drug responses and specificity in these other species.

## Discussion

### Basal and enhanced slowing responses in the Eurhabditids vs. outgroups

**Figure 7:**
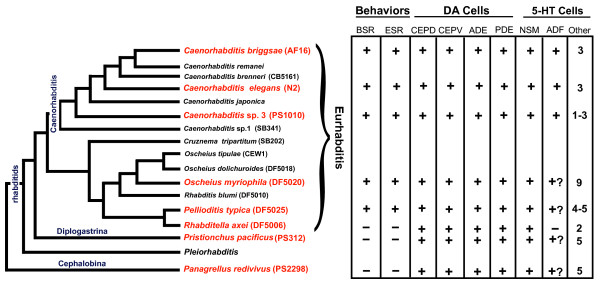
**Mapping behavioral data and neuronal characters on the current nematode phylogeny**. To understand the possible evolution of modulated behaviors and biogenic amine neurons, we overlaid our data on a current phylogenetic tree. We observe that most of the Eurhabditids exhibit both basal slowing and enhanced slowing responses with the exception of *R. axei*. Outside of the Eurhabditids, neither of the other species tested exhibited a basal or enhanced slowing response in our experiments. The number of dopaminergic neurons was essentially unchanged among the different species examined. For the serotonin-immunoreactive neurons, the NSM neurons are conserved across all species; conservation of other cells is less certain. A presumptive ADF neuron is found in several species; overall, the number of serotonergic neurons varies considerably among the nematode species tested.

Most species we tested in the Eurhabditis clade exhibited both basal and enhanced slowing behaviors, with the exception of *Rhabditella axei*. The nematode species outside of the Eurhabditis clade, *Pristionchus pacificus *and *Panagrellus redivivus, *exhibited neither a basal nor enhanced slowing response under the conditions we tested (Figure 7). We must consider possible reasons for the absence of these behaviors in some species, and in the strains we tested. Slowing presumably increases the likelihood of the worms remaining in a location with a desirable food source. Worms that do not slow may not find *E. coli *a desirable food, despite each of these laboratory wild type strains having been raised for many generations on *E. coli *as food. It is unlikely that any of these worms normally feed on *E. coli *in the wild, so it is possible that another bacterium would elicit a BSR. (Or some other edible microbe - *Pristionchus *has been called an 'algivore-omnivore-predator' [18]). Our test of other bacteria with *C. elegans, Pristionchus *and *Panagrellus *(Figure 3) suggests there can be considerable variability in responses to different bacteria. No bacterial strain tested elicited a BSR in *Pristionchus *and *Panagrellus *- both tended to increase their locomotory rate in bacteria. *C. elegans *also failed to slow on the unfamiliar non-*E. coli *strains presented - there was no difference in rate on two pathogenic bacteria (*P. aeruginosa *and *S. marcescens*), and worms sped up on what should be an adequate food source (*B. subtilis*). It is possible that responses would be different if worms were accustomed to the bacteria. *C. elegans *is known to alter its chemotaxis behavior following exposure to pathogenic bacteria [32].

We should note that a purely mechanosensory effect of bacteria on modulating locomotion in well-fed worms - the model proposed by Sawin and colleages [17] - is called into question by our observations of locomotory rates on various bacteria in *C. elegans*. Although both *E. coli *strains elicited slowing, the other bacterial species either had no effect, or caused worms to speed up. All these bacteria are of similar size and shape, and it is difficult to imagine that their mechanical properties are more different from *E. coli *than similar-sized Sephadex beads (which can elicit a BSR [17]). Therefore it seems likely that bacteria elicit more than just a mechanosensory effect, even in well-fed worms. Perhaps in *C. elegans *and other species, chemosensation can override mechanosensory input to prevent slowing in less desirable or unfamiliar food sources.

For the enhanced slowing response, we must ask, how long a period of food-deprivation is sufficient to 'motivate' worms to slow in food? Perhaps we would see a BSR and ESR in the presence of a desirable edible microbe, and an ESR given an adequate period of starvation. What about the normal ecology of the species examined? The laboratory environment may not sufficiently mimic the natural ecology of some species to induce slowing behaviors. In general, the ecology of free-living nematodes and how their natural history influences their behavior is poorly understood (see Kiontke and Sudhaus, 2006 for review [19]). Other environmental conditions that are known to strongly affect behavior include oxygen concentration; thick bacterial lawns can have significantly lower levels of oxygen [40]. The use of very thin lawns in our experiments should reduce the possibility of such a separate influence of bacteria on behavior. But a different oxygen concentration environment, for example, might reveal a BSR and ESR in the other species. Some or all of these worms likely prefer lower oxygen levels; *C. elegans *seems to prefer ~5-12% O_2 _[40].

Our studies suggest that particular forms of BSR and ESR are present in the species in question, and are missing in the others. Therefore, the available molecular phylogeny [3] can suggest that the BSR and ESR we observed may have evolved in the Eurhabditis clade and were perhaps lost in *R. axei *(Figure 7). Perhaps some aspect of behavioral and/or trophic ecology of *R. axei *is different from the other Eurhaditids - despite *R. axei *having been isolated from compost like many of the other species. Worm species lacking a BSR or ESR might have evolved alternate behaviors, such as increased pharyngeal pumping, that optimize foraging without slowing locomotion. We must also consider whether laboratory strains may have lost behaviors that are found in the wild, due to genetic drift and selection in laboratory culture. While considering such a possibility we note that the *C. elegans *laboratory wildtype strain N2 - which has the BSR and ESR - has been raised in the laboratory much longer than any of the other strains we tested, being isolated from the wild in 1956 [41,42].

It is interesting to note that distinct, non-overlapping circuits mediate the BSR and ESR in *C. elegans*. This is seen most strikingly in *C. elegans *mutants lacking dopamine: although there is a complete loss of the BSR, after food-deprivation, the ESR is undiminished [17]. Therefore, under conditions evoking an ESR, there is no contribution in slowing by a BSR. A plausible explanation for this observation is that the separation in the pathways mediating basal slowing versus enhanced slowing behaviors might have evolved at different times. Given such a justification, however, it is interesting to note that among the species we examined, either both responses are seen or neither is seen.

### Evolution of biogenic amine neurons mediating slowing responses in Rhabditids

The basal slowing response in *C. elegans*, presumably present to slow worms in food to increase feeding, is triggered by the mechanical sensation of bacteria by sensory neurons that release dopamine. These four bilaterally paired ciliated neurons appear to be highly conserved in nematodes[43]. Cephalic sensilla were likely present in the nematode stem species, deirids in the stem species of Secernentea and Plectida; postdeirids perhaps only in the stem species of Secernentea (K. Kiontke, personal communication), which includes all the species we examined. Expression of dopamine in likely homologs may also be highly conserved. *Trichinella spiralis*, traditionally considered a 'basal' nematode, has four catecholamine-positive cells with neurites in the cephalic sensilla [43]. The distantly-related plant parasitic nematode *Xiphinema americanum*, also has four FIF-positive cephalic sensilla neurons and 2 possible deirid neurons in locations very similar to those described here [44]. A similar pattern of dopamine-containing neurons to what we report here, but via dopamine immunoreactivity, has also previously been described for *Panagrellus redivivus *[45].

In our study, the presumptive CEP, ADE and PDE homologs all appear to contain dopamine as they do in *C. elegans *(Figure 7). The positions of FIF-positive somata in the head and body were very similar in all worms, and the morphology of processes seen by 5HTP-induced serotonin immunoreactivity was also mostly like that seen in *C. elegans *DA neurons. The most parsimonious explanation is that these are all homologous neurons. The alternative explanation - that different, non-homologous neurons express dopamine in some of the species we examined - requires two events: loss of expression in one cell type, and new expression in another.

It seems likely that the mechanosensory function of cephalic and deirid sensilla neurons is also conserved, despite the lack of conservation of the BSR in all the species we examined. As suggested above, perhaps the type of microbe matters: the mechanical properties of some bacteria (such as *E. coli*) could be insufficient to activate the cells appropriately in some species. Or, the neural circuitry could be different - either by altered connections, or distribution and number of receptors - so that the role of dopamine is changed. In *C. elegans*, dopamine actually plays a complex (and extrasynaptic) role in regulating the BSR: whereas knockout of the *dop-3 *dopamine receptor causes loss of the BSR, in a double knockout mutant for both *dop-1 *and *dop-3 *dopamine receptors, the BSR is partially restored [26]. This indicates that dopamine also inhibits the BSR (promotes locomotion), and that a balance of these antagonistic influences likely fine-tunes the locomotory rate. Therefore, it is easy to imagine the system being biased toward a different effect of dopamine release. Such a shift in the balance of positive and negative influences of dopamine could underlie the increased locomotion of *Pristionchus *on bacteria. In addition, other environmental conditions or the behavioral state of the worm that affects dopamine or its receptors could mean that a BSR will not be seen under the conditions we tested.

The role of serotonin and specific serotonergic neurons in experience-dependent modulation of locomotion in *C. elegans *is less clear-cut than the role of dopamine. Loss of serotonin causes partial loss of the ESR, and ablation of the NSMs only slightly reduces the ESR. Ablation of many other serotonergic neurons along with NSMs does not reduce the ESR more than NSM ablation alone [17]. The clearest effect of serotonin in the ESR is demonstrated by the role of the inhibitory MOD-1 serotonin receptor [26]. It is likely that both serotonin and the neurons that use serotonin have both positive and negative effects on regulation of locomotion, similar to what is seen with dopamine. Serotonin has been demonstrated to both promote and inhibit egg laying in *C. elegans *[46,47]. It is possible that the ESR in *C. elegans *is primarily triggered by serotonin (and perhaps other neurotransmitters) released from the NSM. As with our examination of BSR and dopaminergic neurons, the presence of a given neuron in other species does not correlate with the presence of the behavior. NSMs are clearly recognizable, and highly conserved among these nematodes (and many other free-living species, as reported elsewhere [48]). It is certainly possible that the serotonergic NSMs are required, but not sufficient, for generating an ESR in the species in which we observed it. Other neurons are less well conserved, and difficult to identify definitively as homologous neurons. Although likely ADF homologs are found in many of the species in question, their presence does not correlate with the ESR, and evidence in *C. elegans *suggests they do not play a role in the ESR [32].

Our experiments with the serotonin antagonist mianserin suggest that the ESR, like in *C. elegans*, is serotonin-dependent in *Oschieus myriophila*, although this is less clear in *C. briggsae*. It should be noted, however, that we do not know the specificity of this antagonist with all the biogenic amine receptors in *C. elegans*, let alone in the other species. Furthermore, more recent experiments have shown that mianserin can also affect identified tyramine receptors in *C. elegans *[49,50], although there is no evidence of tyramine or octopamine involvement in slowing behaviors [51]. The role of serotonin in regulating locomotion may have changed in *Caenorhabditis *sp. 3 - the serotonin antagonists mianserin and methiothepin depress the rate of locomotion with or without bacteria, and do not appear to block the ESR. If these agents still work as antagonists (another possibility is that the pharmacology of the receptors has changed so that both these agents act more like agonists), the balance has shifted in *Caenorhabditis *sp. 3 wherein serotonin's normal predominant role may be to increase rather than inhibit locomotion. We may also conclude that evolutionary changes in behavior are less likely to be caused by obvious, gross changes in neurotransmitter expression of neurons, but by more subtle changes in neural circuitry or changes in gene expression in the different species. It has been shown that in certain conserved behaviors, the sensory architecture mediating these behaviors shows marked flexibility during nematode evolution [15,52].

## Conclusions

Therefore, in conclusion the changes we observe in our modulated locomotion studies suggest that some of these changes could occur at the level of the neural circuitry mediating these behaviors. Further understanding of the neural circuitry and the signaling pathways mediating these behaviors could shed light onto how these behaviors evolved.

## Methods

We cultured free-living nematode strains using standard methods for *C. elegans *[53]. Worms were raised at 20°C on NGM plates seeded with the OP50 or HB101 *E. coli *strain (see below). Nomenclature used here conforms to the conventions for *C. elegans *genetics set forth by R. Horvitz and others (1979). Conventions for naming wild-type non-*C. elegans *nematode strains are similar, with each unique isolate receiving a unique strain designation.

### Strains

We used the following worm strains: *Caenorhabditis elegans *(N2) [53], *Caenorhabditis briggsae *(AF16) [54], *Caenorhabditis *sp. 3 (PS1010), *Oscheius myriophila *(DF5020), *Pellioditis typica *(DF5025), *Rhabditella axei *(DF5006) (NYU Rhabditid collection), *Pristionchus pacificus *(PS312) [20], and *Panagrellus redivivus *(PS2298; PS1163) [55]. For *Panagrellus redivivus*, PS1163 was used for studies of dopamine-containing neurons, but PS2298 was used in all locomotory behavior assays. There are no apparent differences in neurons of the two strains [48]. For testing locomotory behavior on other bacterial strains, we chose *E. coli *HB101, *Bacillus subtilis, Pseudomonas aeruginosa *(PA15) and *Serratia marcescens*. All the bacterial strains tested are gram negative except for *B. subtilis*, which is gram positive. Both *Pseudomonas aeruginosa *and *Serratia marcescens *strains are pathogenic for *C. elegans *[56,57]

### Manual counting of body bends

10-12 L4 hermaphrodites or females were picked onto 6 cm NGM plates seeded with HB101 *E. coli *and stored in an incubator at 20°C 16-20 hours prior to the assay; these worms had been continuously cultured on HB101 [17]. Ring plates were also prepared 16-20 hours prior to the assay by spreading 80 μl of HB101 bacteria on 6 cm NGM plates, leaving a circle approximately 1.5 cm in diameter in the center and the edge of the agar unseeded. The plates were incubated overnight at 37°C. For the assay, worms were removed from their overnight cultures using M9. They were rinsed and briefly centrifuged at 6000 rpm to facilitate the transfer of the worms to an assay plate. For 'normal/baseline locomotion', worms were transferred to unseeded 6 cm NGM plates. To test the basal slowing response, worms were transferred to seeded 6 cm ring plates. For 'enhanced slowing', the worms that were used for the 'normal/baseline locomotion' assay were allowed to remain on the unseeded plate for 30 minutes before they were transferred to a ring plate. Worms were allowed to acclimate to the assay plates for 5 minutes, and then the number of body bends/20 seconds was determined for each worm.

### Automated worm tracking and data extraction

Worms tested by automated tracking were continuously cultured on *E. coli *OP50, and tested on OP50. For assaying 'normal/baseline locomotion', 10 cm non-seeded NGM plates were used. To test the 'basal slowing' response, worms were placed on assay plates with a thin lawn of an overnight culture of *E. coli *OP50 [58]. For 'enhanced slowing' studies, worms grown overnight at 20°C on seeded plates with food, were placed on a standard 10 cm NGM plate without food for 30 minutes as described in Sawin et al. [17]. Care was taken to avoid transferring any food from the seeded plates to the assay plates. After 30 minutes, each individual worm was tested for 5 minutes on assay plates containing food.

As previously described [58], 10 cm NGM plates used for recordings were equilibrated to 20°C for 18-20 hours. Approximately one hour before beginning recordings, 600 μl of fresh OP50 overnight culture was spread on each plate to achieve a thin, featureless lawn of food across the entire surface. Excess solution was drawn from the edge with a Pipetman. Food was allowed to dry on the agar surface of a tissue-covered plate until the surface exhibited a matte finish (about 45 minutes), at which time, tissues were replaced by Petri dish lids and plates were ready for use. L4 hermaphrodites or females of each species were picked to fresh seeded plates 16-20 hours prior to recording. Individual worms were transferred to assay plates and the plate placed in a holder on the microscope stage. After two minutes recovery, the worm was located and recording begun using an automated worm tracker and image recorder specially designed for studying worm locomotion [7,58]. Each worm was recorded for five minutes. Data extraction, processing and analysis was done using image processing and analysis software as previously described [7,58]. From each video recording of 5 minutes, we used the middle 4 minutes, and used the software to derive values for frequency of undulations. All incubations and recordings were done in a constant temperature room at 20°C.

### Statistical Analyses

For all behavioral studies, we performed 1-factor ANOVA followed by planned pairwise comparisons made with Scheffè's F-test [59]; all statistical analyses were performed using Excel.

### Serotonin antagonist studies

Behavioral studies were performed as described with the following modification. Approximately 1 hour prior to the assay, mianserin hydrochloride or methiothepin mesylate was added to unseeded and seeded 60 mm NGM plates for a final concentration of 20 μM mianserin hydrochloride or 44 μM methiothepin mesylate (both from Sigma-Aldrich). Worms were transferred to the plates and incubated for 30 minutes at 20°C prior to the start of the behavioral assays.

### Formaldehyde induced fluorescence (FIF)

A simplified version of the FIF technique has been described ([60] and R. Lints, personal communication). A small number of worms were picked directly into a 5 μl drop of 4% paraformaldehyde (PFA) in 0.1 M sodium/potassium phosphate buffer (pH 7.2) on a microscope slide. The PFA solution was wicked away with filter paper leaving dry worms by 5 min of exposure; the slide was then heated to 98°C for 10 min on a metal block. The slide was briefly cooled to room temp, a drop of 100% glycerol added to the worms, and a coverslip was placed over the prep. Worms were viewed with a Chroma 11003v2 Blue/Violet filter set; DA fluorescence had a characteristic blue-green color whereas most background fluorescence was more yellow-green. In all species, the best staining was in young larvae; older larvae and adults more rarely had good FIF staining, and had higher background. For reliable FIF in *Panagrellus*, worms had to be cut open in the 4% PFA solution, suggesting that access in larger intact worms is the key problem.

### 5HTP treatment

Worms were incubated at 20°C for 8-12 hr on 60 mm NGM agar plates containing 5 mM 5-hydroxytryptophan (5HTP, Sigma-Aldrich) and seeded with bacteria. Worms were removed from the plate by washing with M9 buffer, and subsequently processed with the standard anti-serotonin protocol (below). Overall background staining was typically increased in 5HTP-treated preparations.

### Serotonin immunohistochemistry

Rabbit anti-serotonin antibody (antigen: serotonin paraformaldehyde-conjugated to bovine serum albumin) was purchased from Sigma (St. Louis, MO; catalog S4454, lot 091K4831). We have previously tested the specificity of staining with this antiserum in 14 different species of free-living nematode, including all those tested here [48]. Staining is blocked by the antigen, and partially by free serotonin, but not other agents. We have similarly shown no staining in controls with secondary antibody alone [48]. A previously described fixation and staining procedure was used [61], with some modification [31]. Briefly, worms in a mixed-stage population were washed from 60-mm culture plates with M9 buffer, rinsed three times to remove bacteria, and then fixed overnight (ON) at 4°C in 4% PFA in PBS. The worms were rinsed in 0.5% Triton X-100/PBS (TPBS), incubated ON at 37°C in 5% 2-mercaptoethanol/1% TX-100/0.1 M Tris (pH 7.4), rinsed in TPBS, and then incubated for 30 minutes to 4 hours at 37°C in 2000 U/ml collagenase type IV (Sigma) in 1 mM CaCl_2_/1% TX-100/0.1 M Tris, pH 7.4. Following TPBS rinses, the worms were "blocked" for ≥ 1 hour in 1% BSA/TPBS at RT, then incubated ON at RT in 1:100 antiserotonin serum in 1% BSA/TPBS. The worms were rinsed 2× in TPBS, then for 1 hour in 0.1% BSA/TPBS, incubated for 2-4 hours at 37°C with 1:100 TRITC- conjugated goat anti-rabbit IgG, and rinsed briefly several times in 0.1% BSA/TPBS. About 5-10 μl worms from the final preparation were pipetted onto an agarose pad, coverslipped, and viewed with epifluorescence. Worms were also often stained with DAPI to mark nuclei. In some cases this was necessary to determine whether a stained structure was a neuronal cell body (with a DAPI-stained nucleus) or not.

## Competing interests

The authors declare that they have no competing interests.

## Authors' contributions

LR, AS, and SO performed manual locomotion studies, LR, AS and CL performed anti-serotonin staining studies, CL performed FIF and 5HTP/anti-serotonin studies, JS and PWS designed tracker experiments and JS performed automated tracker locomotion experiments, and LR, JS, CL and PWS wrote the paper. All the authors have read and approved the final manuscript.

## Supplementary Material

Additional file 1 **Locomotory rates can be determined manually or by automated tracking**. Two different techniques were used to determine the locomotory behavior of eight species of nematodes: *C. elegans *(N2), *C. briggsae *(AF16), *Caenorhabditis *sp. 3 (PS1010), *Oscheius myriophila *(DF5020), *Pellioditis typica *(DF5025), *Rhabditella axei *(DF5006), *Pristionchus pacificus *(PS312), and *Panagrellus redivivus *(PS2298). The species selected exhibit a sinusoidal pattern of body bends similar to *C. elegans *and are a diverse group of taxa in the rhabditid phylogeny. The species include both gonochoristic and hermaphroditic life histories, so hermaphrodites or females were used. In the first technique, locomotory rates were determined as previously described [17], by manually counting body bends in a 20 second period. In the second technique, an automated worm tracker was used to analyze the different locomotory parameters such as frequency of body bending (Hz) etc. (See Materials and Methods, Additional File 1). For both techniques, animals were cultured on *E. coli*, washed, and then transferred to assay plates. A baseline locomotory rate was determined by placing well-fed animals on assay plates lacking a bacterial lawn. The basal and enhanced slowing responses were measured by transferring well-fed or food-deprived animals, respectively, onto assay plates with a ring-shaped bacterial lawn. Locomotory rates were determined manually for *C. elegans, C. briggsae, Caenorhabditis *sp. 3, *O. myriophila, P. typica *(DF5025) and *R. axei*. The automated tracker was used for all species. Worm velocity determined using the automated tracker was converted to locomotion frequency (body bends/sec or Hz) for comparison purposes. The same slowing trends were observed regardless of the assay method used (Additional File 1), although the manually counted rate of body bends does not directly match the frequency (a distribution) determined by the tracker. Click here for file
