# Supplementary figures and images for: A comparison of experience-dependent locomotory behaviors and biogenic amine neurons in nematode relatives of Caenorhabditis elegans
Source: BMC Neurosci. 2010 Feb 19;11:22. doi: 10.1186/1471-2202-11-22 (PMC2836364; doi:10.1186/1471-2202-11-22)

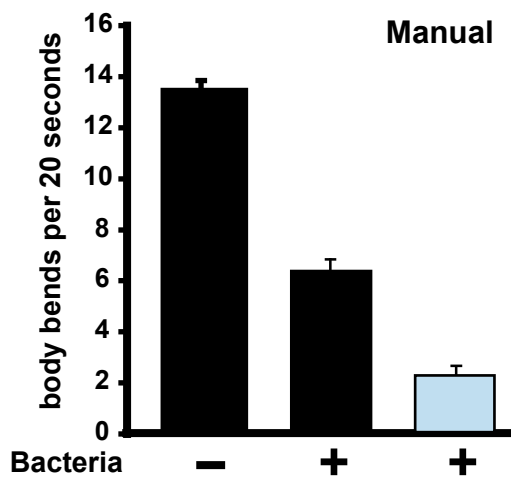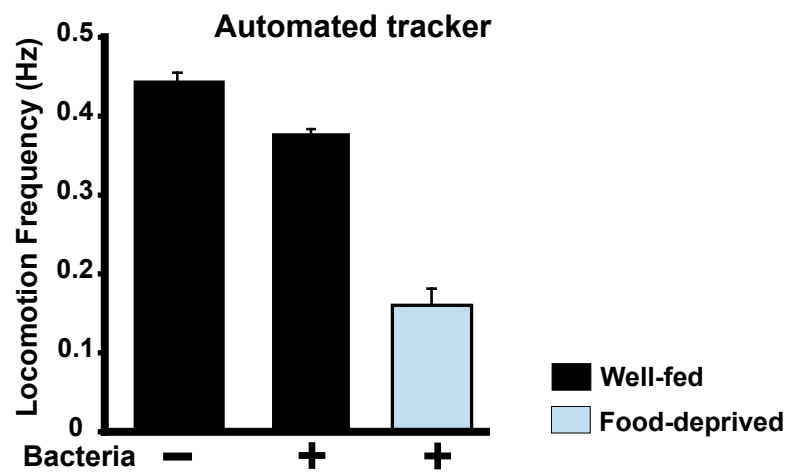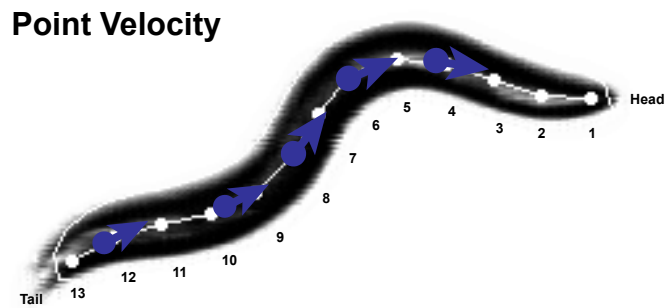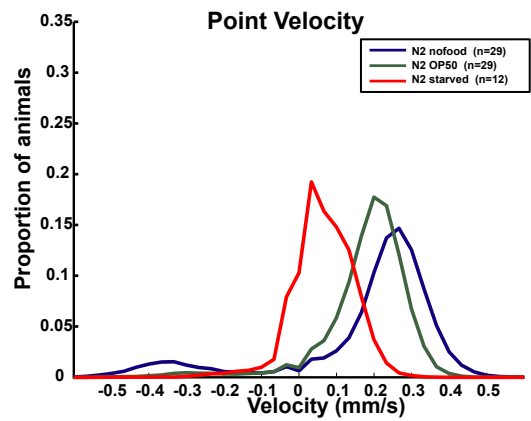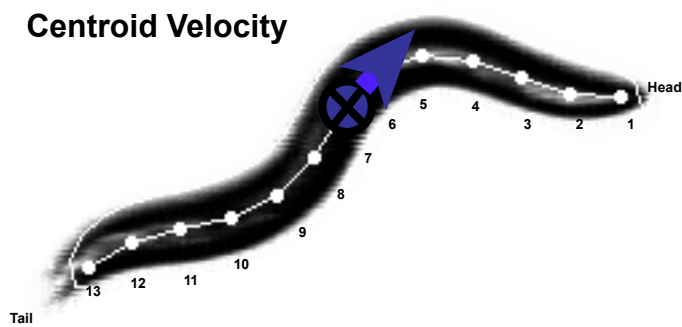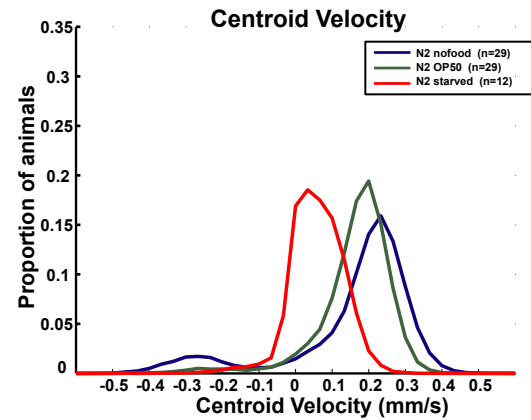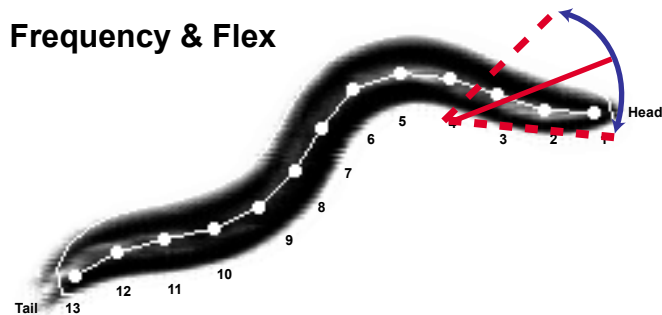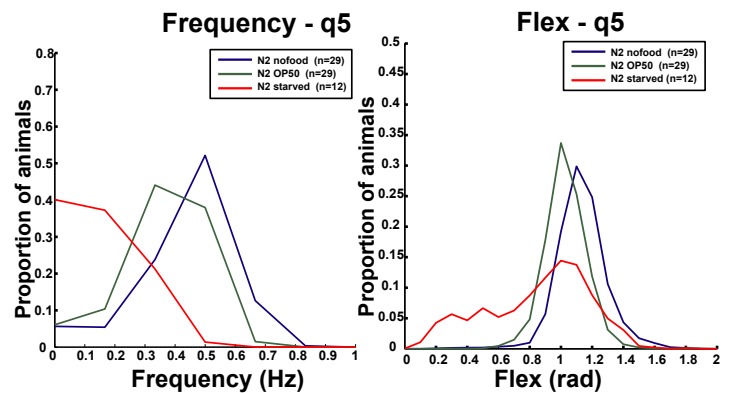

Supplementary Figure 1

Supplement: Additional file 1 — Locomotory rates can be determined manually or by automated tracking. Two different techniques were used to determine the locomotory behavior of eight species of nematodes: C. elegans (N2), C. briggsae (AF16), Caenorhabditis sp. 3 (PS1010), Oscheius myriophila (DF5020), Pellioditis typica (DF5025), Rhabditella axei (DF5006), Pristionchus pacificus (PS312), and Panagrellus redivivus (PS2298). The species selected exhibit a sinusoidal pattern of body bends similar to C. elegans and are a diverse group of taxa in the rhabditid phylogeny. The species include both gonochoristic and hermaphroditic life histories, so hermaphrodites or females were used. In the first technique, locomotory rates were determined as previously described [17], by manually counting body bends in a 20 second period. In the second technique, an automated worm tracker was used to analyze the different locomotory parameters such as frequency of body bending (Hz) etc. (See Materials and Methods, Additional File 1). For both techniques, animals were cultured on E. coli, washed, and then transferred to assay plates. A baseline locomotory rate was determined by placing well-fed animals on assay plates lacking a bacterial lawn. The basal and enhanced slowing responses were measured by transferring well-fed or food-deprived animals, respectively, onto assay plates with a ring-shaped bacterial lawn. Locomotory rates were determined manually for C. elegans, C. briggsae, Caenorhabditis sp. 3, O. myriophila, P. typica (DF5025) and R. axei. The automated tracker was used for all species. Worm velocity determined using the automated tracker was converted to locomotion frequency (body bends/sec or Hz) for comparison purposes. The same slowing trends were observed regardless of the assay method used (Additional File 1), although the manually counted rate of body bends does not directly match the frequency (a distribution) determined by the tracker. [file 1471-2202-11-22-S1.PDF]
